# Hazardous Effects of Titanium Dioxide Nanoparticles in Ecosystem

**DOI:** 10.1155/2017/4101735

**Published:** 2017-03-08

**Authors:** Syed Niaz Ali Shah, Zahir Shah, Muzammal Hussain, Muzaffar Khan

**Affiliations:** ^1^Department of Chemistry, Quaid-i-Azam University, Islamabad, Pakistan; ^2^Department of Chemistry, Tsinghua University, Beijing 100084, China; ^3^Guangzhou Institutes of Biomedicine and Health, Chinese Academy of Sciences, 190 Kaiyuan Avenue, Science Park, Guangzhou 510530, China; ^4^Department of Biochemistry, Faculty of Biological Sciences, Quaid-i-Azam University, Islamabad, Pakistan

## Abstract

Although nanoparticles (NPs) have made incredible progress in the field of nanotechnology and biomedical research and their applications are demanded throughout industrial world particularly over the past decades, little is known about the fate of nanoparticles in ecosystem. Concerning the biosafety of nanotechnology, nanotoxicity is going to be the second most priority of nanotechnology that needs to be properly addressed. This review covers the chemical as well as the biological concerns about nanoparticles particularly titanium dioxide (TiO_2_) NPs and emphasizes the toxicological profile of TiO_2_ at the molecular level in both in vitro and in vivo systems. In addition, the challenges and future prospects of nanotoxicology are discussed that may provide better understanding and new insights into ongoing and future research in this field.

## 1. Introduction

**Figure 1:**
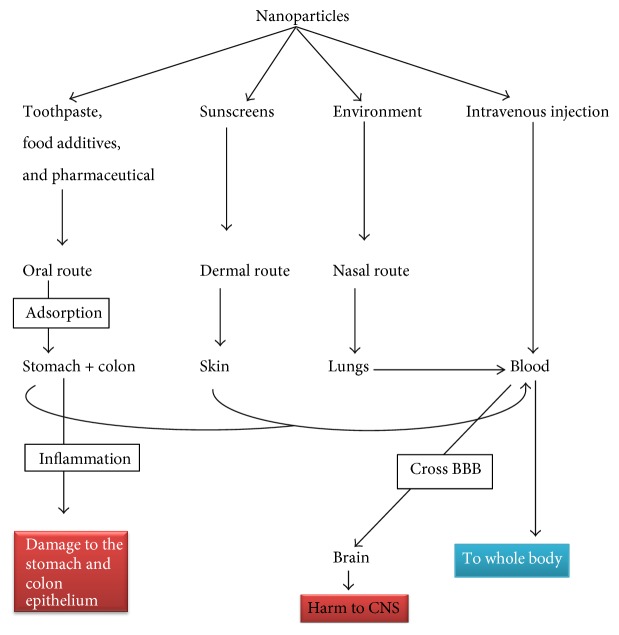
Nanoparticles containing products and their entrance ways into the biological system.

In the last decade, nanoscience has flourished a lot with rapidly advancing nanotechnology and its wider applications [1]. Nanomaterials (NMs) are being and have been exclusively developed and extensively used in a wide variety of products, including medicine, industry, personal care products [2, 3], cosmetics [4], sunscreens [5], toothpastes [6], paints, optics and electronics [7, 8], photocatalysts, antiultraviolet light agents [9], food packaging, medical devices, bandages, clothing, dental restoration material and water treatment facilities [10, 11], antibacterial agents [12], drug delivery systems, artificial organ, and tissue adhesives [13], and for cancer cells apoptosis under UV irradiations (Figure 1) [14]. Moreover, the nanoparticles (NPs) are eminent candidates to overcome drug resistance posed by microorganisms, a major challenge to scientific community [15]. Currently, more than 1000 products or product lines in market contain NPs [16, 17], and it has been estimated that the engineered NMs had reached 2.5 trillion US$ annual profit by 2015 [17]. Nevertheless, the consequently increasing interactions of NPs with biological, chemical, and ecosystems have raised concerns regarding their general and occupational health and safety profiles. The NPs enter the environment and affect both biotic and abiotic components of the ecosystem [18], including human beings [19]. The aquatic ecosystem has also been contaminated with NPs and their negative impacts suppress the immune system of fish and invertebrates [10].

Among the NPs, titanium dioxide NPs (TiO_2_ NPs) are one of the most highly manufactured and widely used in the world [20]. TiO_2_ is a well-known semiconductor and a versatile compound that exists in three crystalline forms, anatase, rutile, and brookite [14, 21], which can only be activated with UV light due to its high band gap energy (3.0 eV for rutile phase and 3.2 eV for anatase phase). The anatase and rutile forms have natural and industrial importance, while the brookite is rarely used. Generally, anatase is more toxic than rutile and, unfortunately, being used abundantly [21, 22]. Many researchers have contributed to the use of TiO_2_ NPs in in vitro and in vivo systems. However, there is a lack of an overall evaluation of their toxicological effects in terms of harmful interactions with the biological and chemical systems and the environment. This review, therefore, specifically intends to provide a brief insight into the toxicological profile of TiO_2_ NPs with respect to biological and ecosystems.

## 2. Confliction about the Toxicological Impacts of TiO_2_ NPs

TiO_2_ is known for long time as “the environmental white knight” due to its limited toxicity [23], inertness, and biocompatibility [8, 24]. The lethal dose at 50% concentration (LD_50_) of TiO_2_ is greater than 10 g/kg [25], and it has been approved as a food additive since 1996 by the Food and Drug Administration (FDA). The FDA and Environmental Protection Agency (EPA) have specified 50 *μ*g/kg body weight/day of nano-TiO_2_ (nTiO_2_) as safe dose for humans (Title 21, volume 1, revised as of April 1, 2014). Moreover, the European Commission's Scientific Committee on Food (SCF), the Joint Expert Committee on Food Additives of the Food and Agriculture Organization/World Health Organization (JECFA), and the European Food Safety Authority (EFSA)'s Scientific Panel on Food Additives, Flavorings, Processing Aids and Materials in Contact with Food have also approved the daily intake of nano-TiO_2_ in general food stuff. Looking from the perspective of potential adverse health effects, several experimental and epidemiological data have demonstrated that TiO_2_ is biologically inactive and physiologically inert, exhibiting relatively low toxicity, thus posing low risk to humans [26]. For example, in a study of chronic toxicity and carcinogenicity, a total of Fischer 344 rats and B6C3F1 mice at concentration of 0, 25000, and 50000 mg TiO_2_/kg diet for 103 weeks (2 years) showed no significant toxicity. In the same study, TiO_2_ coated mica at 0, 1, 2, and 5% in Fischer 344 rats for 130 weeks (2 and half years) had no toxicological or carcinogenic effects [27]. Furthermore, the intraperitoneal injections (IP) of TiO_2_ NPs (5 mg/kg) for 14 days caused no significant adverse effects on mouse kidney [28]. Similarly, both the JECFA and EFSA evaluations of TiO_2_ showed that there is no absorption or tissue storage of TiO_2_, as well as no health hazard effects for occupational workers and public health by Material Safety Data Sheets (MSDS) [8]. In addition, the World Health Organization (WHO)'s Environmental Health states that “titanium compounds are poorly absorbed from the gastrointestinal tract, which is the main route of exposure for the general population” (WHO 1982), and they pose low hazard potential in mammals or aquatic species* (Daphnia magna, Oncorhynchus mykiss)* [29]. Keeping in view the above-mentioned data, it is obvious to accept that the TiO_2_ NPs are health friendly and nontoxic to biological environment.

Contrarily, the Scientific Committee on Consumer Safety (SCCS) has described the genotoxic, carcinogenic, and photosensitization behavior of TiO_2_ NPs (SCCS/1516/13), and several in vitro and in vivo studies have shown the adverse effects of TiO_2_ NPs in biological systems [30, 31]. Recently, Yin et al. [8] have shown that all the molecular sizes and crystal forms (anatase and rutile) of nTiO_2_ may cause phototoxicity [mainly caused by reactive oxygen species (ROS)] under UV irradiations [8] and exert acute toxicity in mice at different dosages of 0, 324, 648, 972, 1296, 1944, or 2592 mg/kg body weight [32]. ROS may further upregulate the inflammatory cytokines and apoptosis-related genes [24, 33, 34], inhibit the heat shock proteins (HSP) [24, 35], and cause neuroinflammation (Figure 4) [36]. The small size (10–20 nm) TiO_2_ NPs may induce oxidative DNA damage, lipid peroxidation, and increased hydrogen peroxide (H_2_O_2_) and nitric oxide production in BEAS-2B cells (human bronchial epithelial cell line) without photoactivation [35, 37]. Collectively, on the basis of above-described data, it seems that there is no clear-cut evidence regarding the safe dose of TiO_2_ NPs and great attention is needed while dealing with these nanomaterials.

## 3. Biological Perspective

NPs, being the advent of nanotechnology, have great impact on the environment. Their production and consumption are increasing day by day, which ultimately has increased the contact chances of NPs with the environment. How do these NPs enter the biological system? What mechanism do they follow? And what are the consequences to the cell viability? To answer these questions, one needs to look very carefully while dealing with NPs in in vivo or in vitro studies as discussed in detail in the next sections.

### 3.1. Biological Uptake of TiO_2_ NPs and Their Entry into the Human Cells

The cellular responses toward NPs depend not only on the properties of NPs, but also on the genetic, transcriptomic, and proteomic landscape of the target cells, imparting different cytotoxic and genotoxic outcomes in various cell types [38]. TiO_2_ NPs enter the human body through several ways, including inhalation, ingestion (food stuffs and daily use materials), skin uptake (through skin lesions), and medical injections [39], and may be distributed to different body organs through circulatory system (Figure 1). After internalization, the TiO_2_ NPs interact with cytoplasmic proteome and bring posttranslational modifications, such as acetylation (A549 cells), by oxidative stress and other mechanisms (Figure 3) [39, 40]. They reach the periregion of nucleus, impede the function of endoplasmic reticulum, and block the nuclear pore or enter the nucleus. Inside the nucleus, they interact with DNA [35] and cause the upregulation of cytokines-, oxidative stress-, and apoptosis-related genes [23, 24, 37]. Meanwhile, the defense system of the cell responds in such a way that the first-line defense is provided by superoxide dismutase (SOD) and catalase (CAT) against oxygen toxicity (ROS), and neutrophils participate against foreign particles (discussed in NETosis pathway in Section 3.2). The transformation of oxy-radicals occurs, such that superoxide radical ^•^O_2_^−^ is dismutated to O_2_ and H_2_O_2_ by catalytic activity of SOD enzyme and, then, CAT converts the H_2_O_2_ into water and oxygen. Oxidative stress (ROS) pathway is one of the mechanisms through which TiO_2_ and Ag NPs exert their toxic effects and disturb the life cycle of* Drosophila* via enhanced ROS generation and DNA damage that lead to related adverse consequences (Figure 3) [7, 41]. In sertoli cells (testicular), the exposure of TiO_2_ NPs (2.5, 5, or 10 mg/kg body weight) may cause severe testicular oxidative damage, apoptosis, ROS generation, and lipid peroxidation. TiO_2_ NPs may also cause suppression of SOD, CAT, glutathione peroxidase (GPx), glutathione S epoxide transferase (GST), glutathione reductase (GR), Cytochrome P450, Family 1, Subfamily B, Polypeptide 1 (Cyp1b1), carbonic anhydrase III (Car3), Bcl-2, acetyl-coenzyme A acyltransferase 2 (Acaa2), and Axin upregulated 1 (Axud1) in mouse testis, while enhancing the expression of apoptotic genes in mouse testis [42]. Moreover, the reverse correlation between ROS generation and reduction of glutathione (GSH) in human hepatocellular carcinoma cell line (SMMC-7721), rat hepatocarcinoma cell line (CBRH-7919), human liver cell line (HL-7702), and rat liver cell line (BRL-3A) has shown the toxicity of TiO_2_ NPs [13].

### 3.2. NETosis Pathway

**Figure 2:**
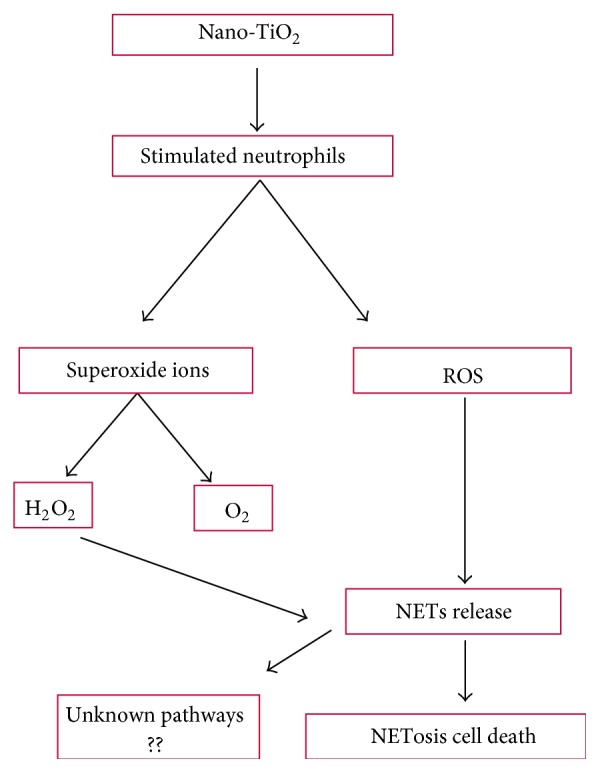
Nano-TiO_2_-induced NETosis cell death pathway.

Neutrophils, the first line of immune defense, have the ability to extrude their DNA (either mitochondrial or nuclear) along with bactericidal, fungal, and protozoal pathogen molecules, thus creating neutrophils extracellular traps (NETs) and releasing them to the extracellular environment. The NETosis pathway is elicited by respiratory burst and ROS generation, causing release of NETs due to formation of superoxide ions. The H_2_O_2_ in phagosome consequently leads to NETs release and NETosis via triggering of the downstream signaling pathways (Figure 2). Exposure of neutrophils to nTiO_2_ may lead to an increased oxidative burst that coincides with NETs release [43]. The NETosis is often accompanied by cell death in order to control and limit extracellular infections, which may otherwise cause complicated human diseases, including sepsis and autoimmune disorders [44, 45].

### 3.3. Apoptosis Mediated by TiO_2_ NPs

Generally, cells remain under constant threats from the cytotoxic and mutagenic effects of DNA damaging agents comprising endogenous (e.g., ROS) and exogenous (such as UV light, ionizing radiations, and other agents like chemicals in foodstuffs, water, or air) or both. Upon DNA damage, the cells undergo either DNA repair or cell cycle arrest leading to apoptosis [46]. Apoptosis is the best described mechanism through which NPs may exert their toxic effects inducing (a) an intrinsic pathway, mediated by mitochondria, or (b) an extrinsic pathway, mediated by death receptors.

**Figure 3:**
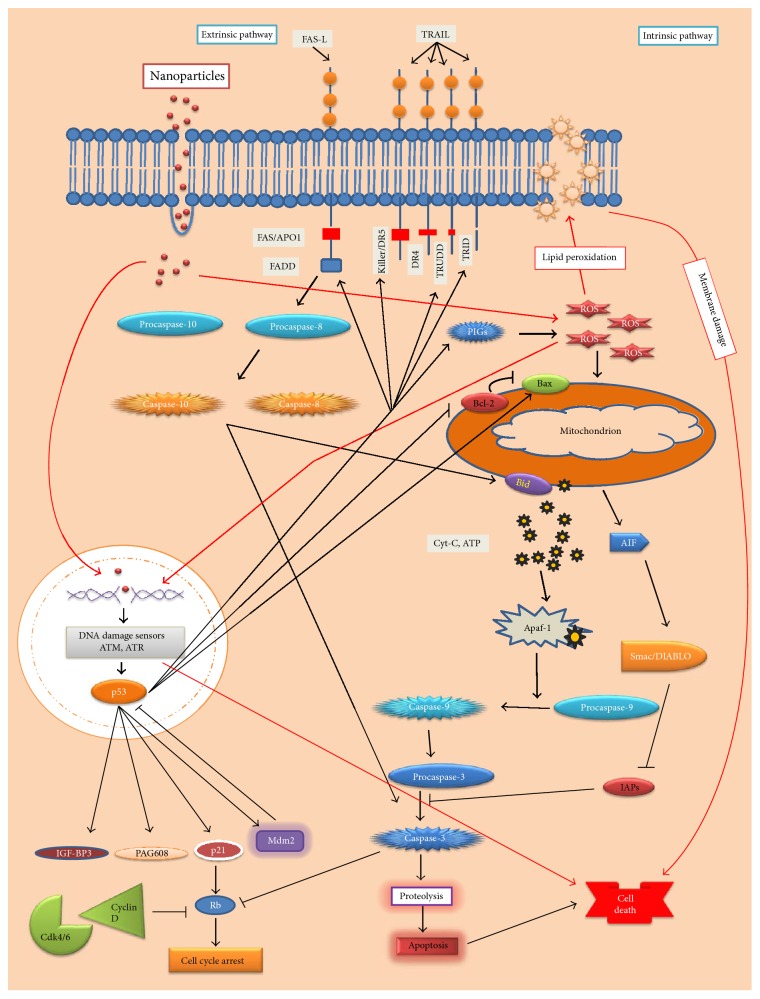
The apoptosis induced by TiO_2_ NPs. The TiO_2_ NPs-induced apoptosis mostly follows the intrinsic pathway. TiO_2_ NPs enter the cell, induce ROS generation, and then enter the nucleus causing DNA damage. The DNA damage is sensed by sensor proteins (ATM/ATR) as a consequence of which p53 is upregulated, which further activates Bax (promoter of apoptosis) and inhibits Bcl2 (inhibitor of Bax).

**Figure 4:**
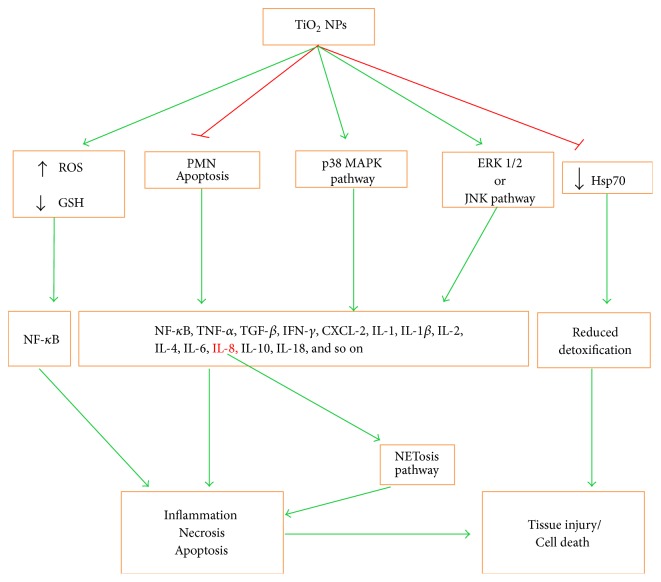
Nano-TiO_2_-induced tissue injury and inflammation. These NPs cause elevation of ROS, decline of GSH levels, inhibition of PMN apoptosis, and tyrosine phosphorylation of p38MAPK and ERK1/2 or JNK. All these induce the production of different inflammatory cytokines that in turn lead to inflammation and consequent necrosis or apoptosis mechanism of cell death. Decreased detoxification due to CYP1A and HSP70 decline also leads to tissue injury or cell death.

TiO_2_ NPs have been shown to induce apoptosis via intrinsic pathway in human bronchial epithelial cell line (BEAS-2B), independent of caspase 8/t-Bid (involved in extrinsic pathway), by enhancing ROS level and proinflammatory responses [28, 33]. During this pathway mitochondrial membrane permeability is enhanced because of caspase-3 release and subsequent PARP cleavage and release of cytochrome C, followed by induction of caspase-9 and caspase-3 (effector caspases) of apoptosis-inducing factor. The genotoxic effect of TiO_2_ NPs upregulates p53 gene that promotes the expression of Bax genes by suppressing Bcl-2 family regulator proteins, thus making an ease for opening the mitochondrial channels and release of cytochrome C (Figure 3) [7, 37, 47]. The accumulation of TiO_2_ NPs in mouse neurons manifests the apoptotic markers such as nuclear shrinkage and chromatin condensation [47]. Furthermore, TiO_2_ NPs may cause the upregulation of oxidative-stress-related genes, including heme oxygenase-1, thioredoxin reductase, glutathione-*S*-transferase, and cytokines such as interleukin- (IL-) 1, IL-2, IL-4, IL-5, IL-6, IL-8, IL-10, IL-12, IL-18, and IL-1*β*, transforming growth factor- (TGF-) *β*, tumor necrosis factor- (TNF-) *α*, and interferon- (IFN-) *γ* (Figure 4), which may cause inhibition of HSP70. The IL-8 gene expression is induced via p38 mitogen-activated protein kinase (MAPK) pathway and/or extracellular signal (ERK) pathway [24, 35]. Similarly, the intragastric exposure of TiO_2_ (2.5, 5, and 10 mg/kg) in mouse may lead to their accumulation in kidneys, inducing necrosis and inflammatory responses (Figure 3) [24].

### 3.4. Phototoxicity and Genotoxicity

TiO_2_ NPs induce phototoxicity upon UV irradiations. They have been shown to induce apoptosis by activating apoptosis-inducing factor (AIF) in human keratinocyte cells [48], as well as in retinal pigment epithelial cells (Figure 3) [2]. Moreover, TiO_2_ NPs have been demonstrated to cause pericardial oedema and premature hatch of* Japanese medaka (Oryzias latipes)* embryos when treated with aqueous suspensions at 0 and 14 *μ*g/mL [49].

The genotoxicity of TiO_2_ NPs attributes to ROS generation and oxidative stress in human epidermal cells, which elicits signal transduction pathways leading to apoptosis or cellular death [33]. They have been shown to induce DNA double-strand breakage in bone marrow and human amnion epithelial (WISH) cells, as well as in mice in a dose-dependent manner, leading to cell cycle arrest [50–52]. The induction of ROS may reduce NADH levels, impairing mitochondrial membrane potential (ΔΨm) and causing mitochondrial dysfunction [53]. The exposure of TiO_2_ and Al_2_O_3_ NPs may also cause genotoxic effects in Chinese hamster ovary after 24 h treatment [54]. The genotoxicity, apoptosis, and mitotic arrest are caused by both nano- and microparticles of TiO_2_ in various tissues of mice [4], as mentioned by SCCS in 2013 (SCCS/1516/13) (discussed in Section 2).

In human lymphocytes, TiO_2_ NPs have been found genotoxic at a dose of 0.25 mM probably by the lipid peroxidation mechanism and at 4 mM to* Allium cepa* [55]. The viability of human epidermal cells was significantly decreased due to DNA damage, micronucleus formation, and reduction in glutathione [14]. They were readily uptaken by A549 cells (carcinomic human alveolar basal epithelial cells) in vitro. However, such rapid uptake was in contrast with a very low oral absorption in a differentiated Caco-2 monolayer system (human epithelial colorectal adenocarcinoma cells) and after oral gavage administration to rats [56]. The calculation of uptake, dispersion, and biological effects of ingested NMs is complicated in vivo due to interindividual differences in the composition, pH, thickness of the mucus layer, gastrointestinal flora, and gastrointestinal passage time [57]. The in vitro studies of NPs interactions are unrealistic and may not indicate the actual fate of NPs, while in in vivo studies, the biological molecules are absorbed on the surface of NPs, changing their biokinetics and the consequent fate of the biomolecules in natural environment [58].

### 3.5. Neurotoxicity

Brain tissues are more susceptible to oxidative stress-induced damage because of high metabolic rate, cellular content of lipids, proteins, and extensive axonal and dendritic networks, and low levels of endogenous scavengers. After exposure of TiO_2_ NPs, the integrity of blood brain barrier (BBB) is badly affected due to persistence of NPs in endothelial cells or via infiltration of immune cells, resulting in breakdown of BBB. In an experimental study, the TiO_2_ NPs have been demonstrated to cause injury to neurons via JNK/p53-mediated-apoptosis and ROS generation, which activated downstream p53/p21 pathway, causing G2/M arrest in in vitro model of dopaminergic neurons (PC12 cell) (Figure 3) [21, 59]. In another study, the TiO_2_ NPs were found to enter the brain via olfactory bulb and reside in the hippocampus region, damaging mitochondria and inducing oxidative stress in rat and human glial cell lines [60]. The anatase nano-TiO_2_ are more toxic to neuronal cells than rutile [21]. Whether these findings have definite neurotoxic implications needs further investigations.

### 3.6. Respiratory Toxicity

The exposures of NMs via inhalation (occupational and/or environmental) may affect the respiratory tract, resulting in an increased risk of lung cancer, fibrosis, blockage of interalveolar areas, and presence of inflammatory cells [17, 61]. The natural and engineered NPs penetrate the lungs through inhalation, reach different body organs via the blood circulatory system [51], and upregulate the inflammatory proteins (MIP and MCP) and genes of MHC class I via Th2-mediated pathway [62]. The IFN-*γ* is preferably released from Th1 cells and induces NPs-triggered cellular immune response along with ROS production in macrophages. They also elicit the expression of GTP-cyclohydrolase I (GCH-I) enzymes, which lead to formation of neopterin, and of indoleamine 2,3-dioxygenase (IDO) (first step is catalyzing enzyme in tryptophan breakdown by kynurenine pathway). The intratracheal exposure of TiO_2_ NPs in mouse may result in their substantial accumulation in lungs, causing bleeding and inflammation [34, 63]. The mutagenic potential of TiO_2_ NPs has been revealed by the treatment of pUC19/lacZ^−^ plasmid with different concentrations of TiO_2_ NPs (average size 30.6 nm) and subsequent transfection of CaCl_2_-induced competent DH5*α* cells, which showed loss of transfection efficacy of the plasmid in comparison to untreated ones [64].

In a cytoplasmic proteome study involving human monocyte-derived macrophages, the abundancies of chloride intracellular channel protein 1, cathepsin D, and lysine acetylation were observed after exposure to nTiO_2_ [40]. Recently, Sheng et al. [65] have demonstrated the significant alterations in the expressions of 1041 genes involved in different types of processes, including immune/inflammatory responses, apoptosis, oxidative stress, stress responses, metabolic processes, ion transport, signal transduction, and cell proliferation/division and translation, in mice spleen [65].

TiO_2_ also caused lung cancer in rats after oral administration of 160 and 33 nm particles at doses of 40, 200, and 1000 mg/kg body weight [4]. The ultrafine TiO_2_ (UF-TiO_2_), less than 100 nm in diameter, induced pulmonary fibrosis, lung tumor, and genotoxicity in rats [66, 67]. Similarly, the NMs may also cause damage to liver cells during cleansing of toxins and pollutants in body. Furthermore, the TiO_2_ NPs may cause hepatotoxicity in human hepatocellular carcinoma cell line (SMMC-7721), human liver cell line (HL-7702), rat hepatocarcinoma cell line (CBRH-7919), and rat liver cell line (BRL-3A), which may be associated with changes in cell morphology, increased intercellular ROS production, and decreased GSH levels at 0.1–100 *μ*g/mL [13].

### 3.7. Aquatic Nanotoxicity

The in vitro studies have raised concerns about the toxicity of TiO_2_ NPs in mammalian, but there are limited data on ecotoxicity to aquatic organisms. The heaping of NPs to sewage increases due to their excessive use in industry and commerce [68]. The engineered nanoparticles (ENPs) intermingle with various toxins, including metals in sediments and water phase, making agglomerates and resides [69], and causing damage to aquatic organisms [55]. The exposure of adult zebra fish to 1.0 mg/L TiO_2_ (both NP and bulk) for 21 days has been shown to lower the number of viable embryos [70] and inhibit the growth of goldfish* (Carassius auratus)* [71]. Similarly, the exposure of nTiO_2_ suspensions (100 and 200 mg/L) to carp (*Cyprinus carpio*) may cause a decrease in SOD, CAT, and POD, while inducing a significant increase in LPO levels in the liver [72]. The combined exposure of anatase and rutile NPs to freshwater microalgae,* Chlorella* sp., at 0.25, 0.5, and 1 mg/L under UV irradiations has been demonstrated to reduce the cell viability and chlorophyll content [22]. TiO_2_ has also adverse impacts on the survival, growth, and reproduction of* D. magna*. It has been determined that exposure of anatase (21 nm) particles is more toxic to* D. magna* as compared to anatase (250 nm) and rutile (500 nm) particles [73]. Therefore, the study of adverse effects of various NMs on aquatic species is necessary to assess their potential environmental hazardous effects.

## 4. Interactions of NPs in Ecosystem

The clean air is not only of scientific, environmental, and physiological importance but a basic need for living a healthy life. The chemicals and biological attacks may pose risk to human health and environment [74]. In this regard, the dangers of NPs to human health and environment have increased due to the prompt growth in nanotechnology. The adsorption of noxious pollutants on NPs has been extensively studied. In environment, the NPs always amalgamate with other pollutants. The interactions between conventional pollutants with NPs and their impact on environmental components are little considered. The heaping of NPs to sewage increases due to their excessive use in industry and commerce [68]. The chance of association of organic materials, including toxicants, increases with the aggregation of NPs in water. Hence, the bioavailability of these materials is altered. Thus, extra toxicological concerns are needed in presence of NPs [75].

The workers involved in the production of TiO_2_ NPs may have significant risk on cytotoxicity response at relatively high airborne concentrations of anatase TiO_2_ NPs [76]. Widespread use of nTiO_2_ may intensify the threat of combined exposure of nTiO_2_ with other environmental pollutants. The mixing of different compounds may bring astonishing toxic effects, even if the toxicities of the individual compounds are well known. For example, when bisphenol A (BPA) combines with nTiO_2_, it facilitates the movement of nTiO_2_ into exposed cells, causing synergistic toxicity by oxidative stress, inducing DNA double-strand breaks and micronuclei formation [77]. The growth inhibition of fresh water algae* (Pseudokirchneriella subcapitata)* was increased by the interaction of Cd(II) species with TiO_2_ [78]. Similarly, the adsorption of Cd(II) onto nTiO_2_ was enhanced by coating humic acid (HA) on nTiO_2_ [79]. The anatase NPs are superior sorbents than activated carbon and other metal oxide NPs [80]. The TiO_2_ and Al_2_O_3_ NPs enter the Chinese hamster ovary (CHO-K1) cells through endocytosis and attack on lysosomal and mitochondrial activities, thus causing cytotoxicity and genotoxicity as well as a decrease in cell viability [54].

The hydroxylated fullerenes/C60 (OH) 24 exert synergistic stimulative effect on genes related to circadian rhythm, vesicular transport, kinases, and immune responses in zebrafish embryos [81], while the presence of nitrite with TiO_2_ enhances the induction of apoptosis-related genes via NO signaling pathway [48].

## 5. Chemical Perspective

From chemical perspective, TiO_2_ NPs show phototoxic effects upon UVA irradiations. Upon photon energy absorption, the electrons of the NPs jump from valence band to the conduction band, leaving the valence band holes. Hydroxyl radicals (^•^OH) are produced when valence band holes take electrons from water or hydroxyl ions and other ROS such as singlet oxygen (^1^O_2_) and superoxide (^•^O_2_^−^) are also produced by different mechanisms. Free radicals (^•^OH and carbon centered free radicals) are also generated in dark. The generated ROS may be genotoxic or cytotoxic, affecting cell viability (Figure 3). Hence, TiO_2_ NPs are toxic to living system both in the presence and absence of light via generation of free radicals [2].

## 6. Effect of Exposure Time and Dose on Toxicity of TiO_2_ NPs

The primary particle size (the size of particle at the time of injection) of TiO_2_ NPs is not as important as that of secondary particle size (the size of particle after agglomeration) for in vivo toxicity. Likewise, the physicochemical characteristics and time of exposure of NPs before the toxicological study are important [82]. The dietary exposure of nTiO_2_ for 3 or 14 days may cause hazards to the terrestrial invertebrates [83]. Intratracheal instillation to rats with 0.5, 5, or 50 mg/kg of 5, 21, and 50 nm TiO_2_ primary particles, respectively, has been demonstrated to exhibit dose-dependent toxic responses. In the same way, intraperitoneal injection of TiO_2_ NPs (5 mg/kg) for 14 days did not have considerable effect on mouse kidney and the nephric dysfunction; however at doses of 50, 100, and 150 mg/kg body weight, it significantly induced inflammatory response and abnormal functions of kidney in mice.

Short-term exposure of TiO_2_ NPs may have low-to-medium ecological hazards on zebrafish [23]. Nano-TiO_2_ exposure for 3 h causes highest production of ROS in cytoplasm while at 24 h exposure ROS is only produced in perinuclear region due to aggregation [35].

Nano-TiO_2_ accumulation occurs around the nucleus for up to 25 days in retinal pigment epithelial cells after a single low-level long-term exposure [2]. The cytotoxicity in normal liver and carcinomatous liver cells of either rat or human increases as the time of exposure increases, even a low concentration of nTiO_2_ may induce higher toxicity with increase in time of exposure [13]. In long-term exposure, TiO_2_ NPs may cause pronounced adverse effect (growth inhibition and loss in liver weight) on zebrafish in time- and dose-dependent manner in vivo. It has also been shown that TiO_2_ NPs exposure for 6 months to zebrafish may elicit pronounced toxic consequences like organ injury, behavior alterations, mortality, and organ distribution at higher concentration [23]. Furthermore, at long-term exposure, TiO_2_ accumulates in the cell and causes toxic effects which are not evident at short-term exposure [84]. Several cell lines exposed to higher concentrations (100 *μ*g/mL of TiO_2_) may exhibit morphological changes such as cell shrinkage or nuclear condensation [35]. The exposure of differentiated murine J774.2 macrophages to 1 *μ*g/mL concentration may have no considerable effects on cell proliferation, while at concentration 10 *μ*g/mL it may exhibit significant cytotoxic effects [12]. Unnithan and colleagues have shown that fine nano-TiO_2_ (~20 nm) at 40 mg/kg cause biochemical perturbations in Wistar rats [85]. Conclusively, the NPs even at their noncytotoxic doses may have pathophysiological concerns [28].

## 7. Effect of Size and Shape on Toxicity of TiO_2_ NPs

The major physicochemical properties to evaluate the toxicity are size, shape, surface area, phase, composition, coating, nature of surface, and agglomeration of NPs [16, 86]. The size and surface area of NPs may be responsible for their toxicity, but most of the studies do not reveal the relationship between physiochemical characteristics of NPs and their toxicity [82]. For example, 25 nm anatase and 31 nm anatase/rutile show greater phototoxicity than 142 nm anatase and 214 nm rutile NPs [2]. All the sizes and crystal forms (anatase and rutile) of TiO_2_ NPs exert toxic (phototoxic) effects on human skin keratinocytes under UVA irradiations in a dose-dependent way. The smaller size nTiO_2_ may cause greater cytotoxicity than larger size NPs, and anatase form may show more phototoxicity than rutile [8, 84]. Furthermore, the NPs (rod and sphere) of smaller size show higher toxicity than larger particles. Moreover, the nanorods exhibit more toxicity than spherical particles having the same size and surface area, showing the contribution of shape toward cytotoxicity [16]. The Ag (20 and 200 nm) and TiO_2_ (21 nm) NPs are significantly taken up by human epithelial, hepatic, and undifferentiated monocyte cells, resulting in decline of metabolic activation and cell death enhancement [87].

## 8. Conclusion and Future Perspectives

Concerning the biosafety of nanotechnology, nanotoxicity is going to be the second most priority of nanotechnology. The different responses toward the NPs in ecosystem may be very complex and diverse, involving a variety of parameters, demonstrating their difficult environmental fate [88]. In addition, the environmental hazards of ENPs can be documented by knowing their behavior and fate in the natural aquatic system [89].

To date no product (medicinal or food stuff) is available with 100 percent purity and efficiency, but for the safer use of nanosized particles with no or minimal hazardous effects on environment, the detailed understanding about their sources, interactions with environment, biodegradability, and possible risk assessment are utmost requirement prior to use. In addition, the interactions of NPs with biological molecules and their adverse effects need to be fully understood prior to their approval in clinical trials.

The cellular responses and toxicity produced by TiO_2_ NPs depend on the surface/mass ratio, purity, crystallinity, surface reactivity, adsorbed groups, coatings, solubility, shape, size [7, 54], zeta potential, and dispersion or propensity to agglomerate or aggregate in different media [90]. These parameters need to be considered for the safer use of NMs. The undefined health and environmental features of TiO_2_ NPs due to its widespread use are necessary to be managed by a systematic, coherent, and tested foundation. Therefore, the regulatory health risk assessment of such particles may be mandatory for the safe use of NMs in consumer products and medicines, including the potential effects on reproduction and fertility.

## Abbreviations

NMs: Nanomaterials
NPs: Nanoparticles
TiO_2_ NPs: Titanium dioxide NPs
LD_50_: Lethal dose at 50% concentration
FDA: Food and Drug Administration
EPA: Environmental Protection Agency
nTiO_2_: Nano-TiO_2_
SCF: European Commission's Scientific Committee on Food
JECFA: Joint Expert Committee on Food Additives of the Food and Agriculture Organization/World Health Organization
EFSA: European Food Safety Authority
MSDS: Material Safety Data Sheets
WHO: World Health Organization
SCCS: Scientific Committee on Consumer Safety
ROS: Reactive oxygen species
HSP: Heat shock proteins
H_2_O_2_: Hydrogen peroxide
SOD: Superoxide dismutase
CAT: Catalase
GPx: Glutathione peroxidase
GST: Glutathione S epoxide transferase
GR: Glutathione reductase
NETs: Neutrophils extracellular traps
TGF-*β*: Transforming growth factor-*β*
TNF-*α*: Tumor necrosis factor-*α*
IFN-*γ*: Interferon-*γ*
MAPK: Mitogen-activated protein kinase
AIF: Apoptosis-inducing factor
BBB: Blood brain barrier
IDO: Indoleamine 2,3-dioxygenase
UF-TiO_2_: Ultrafine TiO_2_
ENPs: Engineered nanoparticles
BPA: Bisphenol A
HA: Humic acid
CHO-K1: Chinese hamster ovary
^•^OH: Hydroxyl radicals
^1^O_2_: Singlet oxygen
^•^O_2_^−^: Superoxide.
